# Diffuse PRAME Expression Is Highly Specific for Thin Melanomas in the Distinction from Severely Dysplastic Nevi but Does Not Distinguish Metastasizing from Non-Metastasizing Thin Melanomas

**DOI:** 10.3390/cancers13153864

**Published:** 2021-07-31

**Authors:** Maximilian Gassenmaier, Matthias Hahn, Gisela Metzler, Jürgen Bauer, Amir Sadegh Yazdi, Ulrike Keim, Claus Garbe, Nikolaus Benjamin Wagner, Stephan Forchhammer

**Affiliations:** 1 Department of Dermatology, Eberhard Karls University of Tübingen, 72076 Tübingen, Germany; matthias.hahn@med.uni-tuebingen.de (M.H.); metzler@zentrum-dermatohistologie.de (G.M.); ulrike.keim@med.uni-tuebingen.de (U.K.); claus.garbe@med.uni-tuebingen.de (C.G.); Stephan.Forchhammer@med.uni-tuebingen.de (S.F.); 2 Dermatologie in Stuttgart, 70173 Stuttgart, Germany; mail@j-bauer.de; 3 Department of Dermatology and Allergology, University Hospital RWTH Aachen, 52074 Aachen, Germany; ayazdi@ukaachen.de; 4 Department of Dermatology, Venereology and Allergology, Cantonal Hospital St. Gallen, 9007 St. Gallen, Switzerland; nikolaus.wagner@gmx.de

**Keywords:** PRAME, immunohistochemistry, melanoma, metastasis, prognosis, nevus, dysplastic nevus, histopathology

## Abstract

**Simple Summary:**

Histological diagnoses within the spectrum from moderately dysplastic nevi to thin melanomas are neither accurate nor reproducible, emphasizing the need for more objective supplemental immunohistochemical markers. PRAME immunohistochemistry aids in differentiating unequivocal melanomas from unequivocal nevi. The aim of our study was to determine whether PRAME IHC also allows differentiation of severely dysplastic nevi from thin melanomas and whether PRAME is of prognostic significance in thin melanomas. We studied 70 thin melanomas, of which 35 metastasized and 35 did not metastasize and 35 severely dysplastic nevi. We found that diffuse PRAME expression was highly specific but only moderately sensitive for thin melanomas. Melanomas and severely dysplastic nevi with PRAME immunoreactivity had different staining patterns. Most Melanomas demonstrated diffuse PRAME staining of intraepidermal and dermal melanocytes while most severely dysplastic nevi showed a decreasing gradient with depth. PRAME did not allow for the differentiation of metastasizing and non-metastasizing melanomas.

**Abstract:**

Background: PReferentially expressed Antigen in MElanoma (PRAME) immunohistochemistry is increasingly used as diagnostic adjunct in the evaluation of melanocytic tumors. The expression and prognostic significance of PRAME in melanomas ≤1.0 mm and its diagnostic utility in the distinction from severely dysplastic compound nevi (SDN) have not been studied. Methods: We investigated and compared the immunohistochemical PRAME expression in 70 matched thin metastasizing and non-metastasizing melanomas and 45 nevi from patients with long-term follow-up (35 SDN and 10 unequivocally benign compound nevi). Results: Diffuse PRAME staining in >75% of lesional epidermal and dermal melanocytes identified 58.6% of thin melanomas but did not distinguish metastasizing from non-metastasizing melanomas (*p* = 0.81). A superficial atypical melanocytic proliferation of uncertain significance, in which the final diagnostic interpretation favored a SDN was the only nevus with diffuse PRAME expression (1/45). Melanomas and SDN with PRAME immunoreactivity exhibited different staining patterns. Most melanomas (67.6%) showed uniform PRAME expression in the in situ and invasive component, whereas most SDN (81.0%) showed a decreasing gradient with depth. Conclusion: Diffuse intraepidermal and dermal PRAME staining is highly specific for melanomas in the distinction from SDN. PRAME expression is not a prognostic biomarker in melanomas ≤1.0 mm.

## 1. Introduction

PReferentially expressed Antigen in MElanoma (PRAME) belongs to the group of cancer testis antigens (CTA) and its eponymous expression in melanomas may provide an immunohistochemical aid in the diagnosis of melanocytic lesions [1]. Lezcano et al. showed that diffuse PRAME expression >75% of lesional cells is highly specific and sensitive for non-spindle cell primary melanomas [1] and lymph node metastases [2]. However, subsequent studies from other groups found lower PRAME expression in both primary [3,4,5,6] and metastatic melanomas [7], calling into question a generally applicable cut-off for melanomas. PRAME expression is not specific to melanoma but is also found in many other solid cancers [8,9] as well as lymphomas [10] and leukemias [11]. In addition, PRAME is useful as a prognostic biomarker, with high expression levels associated with both poor prognosis (uveal melanoma [12], breast cancer [13], neuroblastoma [14], diffuse large B-cell lymphoma [10]) and favorable prognosis (acute myeloid leukemia [11]). The prognostic significance of PRAME in cutaneous melanomas remains to be clarified, as is the immunohistochemical PRAME expression in thin melanomas and severely dysplastic compound nevi (SDN).

Thin melanomas (≤1.0 mm) represent by far the largest subgroup of all melanomas [15] and are of particular interest, as the histological differentiation between a SDN and a thin melanoma poses a frequent diagnostic dilemma. An observer accuracy and reproducibility study has shown that histological diagnoses within the spectrum from moderately dysplastic nevi to thin melanomas are neither accurate nor reproducible, emphasizing the need for more objective supplemental molecular or immunohistochemical markers [16]. In addition, the incidence of thin melanomas (≤1.0 mm) has risen substantially in recent decades and there are concerns, that overdiagnosis due to increased diagnostic scrutiny and not an increase in the incidence of disease has contributed to this trend [15,17,18]. Notwithstanding their overall good prognosis, thin melanomas constitute a potentially lethal disease and contribute substantially to melanoma mortality [15]. Thus, an immunohistochemical marker that distinguishes “true” melanomas with metastatic potential from indolent melanomas and melanoma simulators would be of great help.

In this study, we investigated the expression and prognostic significance of PRAME expression in thin melanomas and assessed its diagnostic utility in the distinction from SDN.

## 2. Materials and Methods

A total of 70 melanomas and 45 melanocytic nevi with follow-up were retrieved from the archives of the Department of Dermatology, University Hospital Tübingen. Patients with thin melanomas (≤1.0 mm Breslow thickness) and initial diagnosis between 2005 and 2020 were searched in the German Central Malignant Melanoma Registry and 35 patients with metastasizing melanomas (MM) and available paraffin-embedded material were identified. Patients with thicker second primary melanomas were excluded. The group with MM was compared with an equally sized control group with non-metastasizing melanomas (NMM). The patients in this group were matched for Breslow thickness (within a 0.1 mm range), sex, age (within a 10-year range), and site (head and neck, trunk, upper extremities, lower extremities). These two groups were further compared with a third group of 35 patients with SDN. The diagnosis of SDN was based on the presence of certain cytologic and architectural criteria according to the World Health Organization classification of skin tumors [19]. These comprised melanocytes with enlarged or hyperchromatic nuclei, prominent nucleoli, and variation in nuclear size and shape in a larger minority of cells. Architectural features included focal but not extensive pagetoid spread, irregular and/or dyscohesive nests of intraepidermal melanocytes, and increased density of non-nested junctional melanocytes. In addition, PRAME staining was analyzed in 10 unequivocally benign compound nevi. All histological diagnoses were based on the original assessment of one or more dermatopathologists experienced in the evaluation of melanocytic tumors (G.M., A.Y., J.B., S.F.). Four ambiguous tumors, in which the final diagnosis favored either an early-stage melanoma or SDN, were included to compare the final diagnostic interpretation with the immunohistochemical PRAME expression and clinical outcome in a real-world setting. Extreme borderline tumors, such as a superficial atypical melanocytic proliferation of uncertain significance (SAMPUS), were not the main focus of this study because the diagnostic assessment of these lesions is exceedingly difficult even with molecular testing. In addition, there is no assurance that a given diagnosis is biologically correct, as these tumors usually do not metastasize if they have been completely excised [20].

Immunohistochemistry (IHC) was performed with antibodies to Ki-67 (dilution 1:100, clone MIB-1, Dako, Santa Clara, CA, United States) and PRAME/Melan-A double staining with antibodies to Melan-A (dilution 1:150, clone A103, Dako) and PRAME (dilution 1:50, clone QR005, DCS, Hamburg, Germany) on an automated immunostainer (Leica Bond-MAX, Leica Biosystems, Wetzlar, Germany) using a brown chromogen (DAB) for PRAME and a red chromogen (FastRed) for Melan-A for a more accurate PRAME quantification and enhanced identification of dermal melanocytes. The percentage of PRAME immunoreactive cells was scored as previously described [1] (0: no staining at all, 1+: 1–25%; 2+: 26–50%; 3+: 51–75%; 4+: >75% positive cells). PRAME staining of any intensity in >75% of epidermal and >75% dermal melanocytes was interpreted as diffuse. Sebaceous glands on the examined slides and a PRAME positive melanoma were used as positive control. PRAME expression was independently quantified by two authors (M.G. and S.F.) in the epidermal and dermal melanocytic component without knowledge of the histological diagnosis and the clinical course. In case of discrepant findings, consensus was achieved together with a third reviewer (M.H.). We decided to quantify PRAME staining in the epidermal and dermal portions of the melanocytic lesions separately, because early invasive melanomas often have an extensive junctional, but only a small invasive component and the latter would not be assessed representatively by quantifying the entire lesion. In addition, PRAME is known to stain the intraepidermal component in some nevi but then shows a decreasing gradient toward depth [1].

Statistical calculations were performed with IBM SPSS version 26 and *p*-values < 0.05 were considered as statistically significant. Interrater concordance regarding diffuse vs. non-diffuse PRAME staining was calculated using Cohen’s kappa (κ). Survival rates were estimated according to Kaplan–Meier and compared with the log-rank test.

## 3. Results

### 3.1. Melanomas

Seventy primary cutaneous melanomas ≤1.0 mm, of which 35 metastasized and 35 did not metastasize, were compared in a case-control study. Patient age ranged from 30 to 91 years with a mean of 62 years. The male to female ratio was 1.5 to 1. Lesions were predominantly localized on the trunk (40.0%), followed by the head and neck (28.6%), lower extremities (20.0%) and upper extremities (11.4%). Cases included 48 superficial spreading melanomas, 16 lentigo maligna melanomas, 3 acral lentiginous melanomas, 2 nevoid melanomas, and 1 spitzoid melanoma. The mean Breslow thickness was 0.7 mm (range 0.3–1.0) and did not differ significantly between melanomas with and without regression (mean 0.7 mm, range 0.3–1.0 vs. mean 0.7 mm, range 0.4–1.0; *p* = 0.81). Mean follow-up was 65 months (range 5–139) for patients with MM and was 99 months (range 31–153) for patients with NMM. The clinicopathologic features of the analyzed melanomas and SDN are summarized in Table S1.

**Figure 1:**
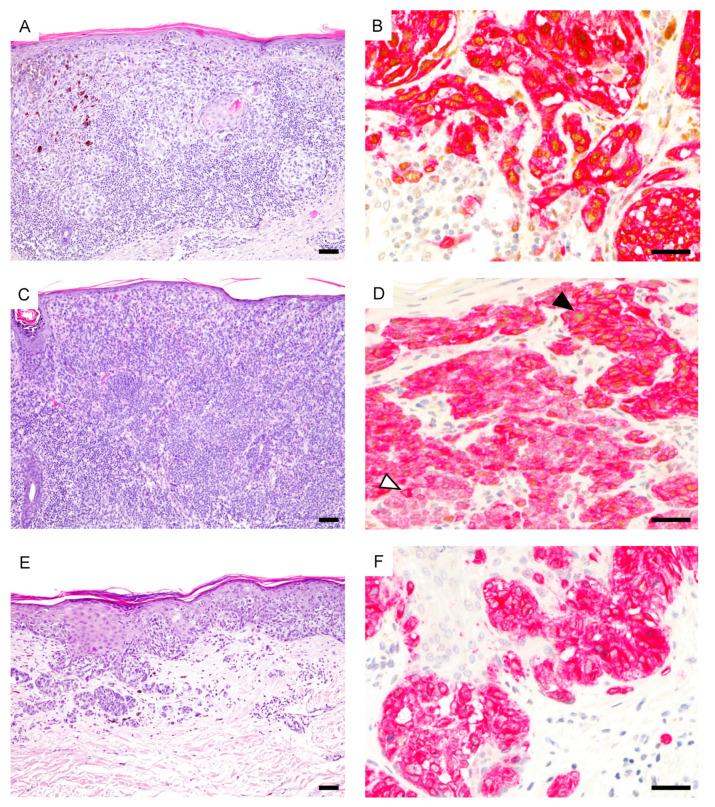
Spectrum of PRAME expression in thin melanomas. (**A**) Superficial spreading melanoma (hematoxylin and eosin (H&E)). (**B**), Diffuse PRAME expression both in the in situ and invasive component (PRAME, brown; Melan-A, red). (**C**) Metastasizing nevoid melanoma (H&E). (**D**), Diffuse junctional PRAME expression (black arrowhead) but predominantly negative dermal melanocytes (white arrowhead) (PRAME, brown; Melan-A, red). (**E**), Superficial spreading melanoma (H&E). (**F**), Melanocytes are PRAME negative (PRAME, brown; Melan-A, red). Abbreviation: PRAME, PReferentially expressed Antigen in MElanoma. Scale bar 100 µm (**A**,**C**,**E**), 50 µm (**B**,**D**,**F**).

**Figure 2:**
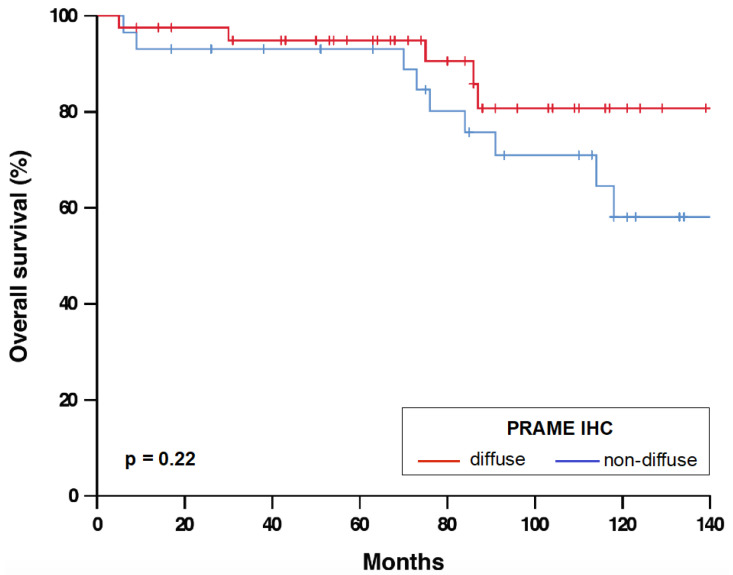
Overall survival of melanoma patients with diffuse und non-diffuse PRAME expression. *n* = 70. *p*-value refers to log-rank test. Abbreviations: IHC, immunohistochemistry. PRAME, PReferentially expressed Antigen in MElanoma.

The results of PRAME IHC are summarized in Table 1 and Figure 1. In total, 41/70 melanomas (58.6%) showed diffuse PRAME staining (PRAME score 4+) in the in situ and invasive component. Of 68 melanomas with PRAME immunoreactivity, 67.6% showed equal PRAME expression in the epidermal and dermal portion and most remaining melanomas (29.4%) weaker dermal expression. Diffuse PRAME expression did not allow differentiation of MM from NMM (*p* = 0.81; Table 2) and was not a prognostic biomarker in melanomas ≤1.0 mm (Figure 2).

**Figure 3:**
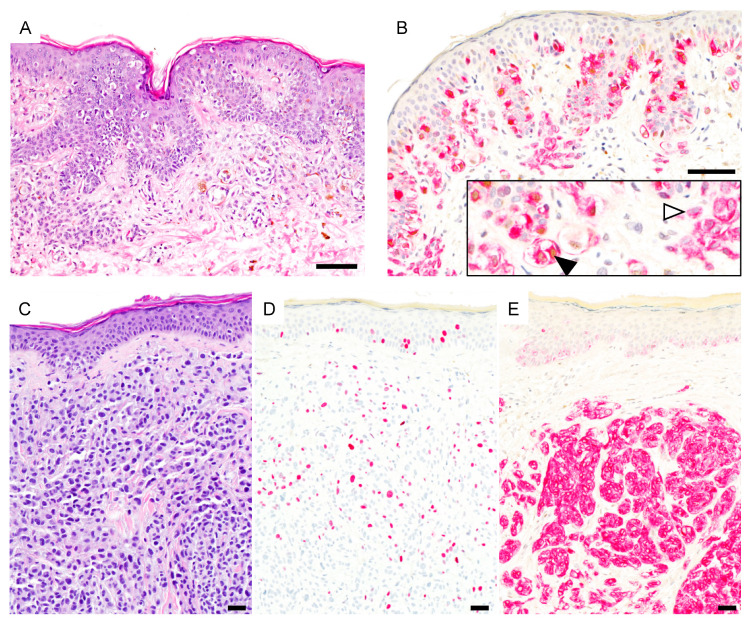
PRAME expression in ambiguous melanocytic lesions. (**A**), Early invasive nevus-associated melanoma (H&E). (**B**), Atypical intraepidermal and dermal melanocytes are PRAME positive (black arrowhead) while the associated nevus is PRAME negative (white arrowhead) (PRAME, brown; Melan-A, red). (**C**), Metastasizing nevoid melanoma (H&E). Array comparative genomic hybridization showed a loss of whole chromosome 9 (not shown). (**D**), Ki-67 staining reveals increased proliferation. (**E**), Melanocytes are PRAME negative (PRAME, brown; Melan-A, red). Abbreviation: PRAME, PReferentially expressed Antigen in MElanoma. Scale bar 50 µm.

There were 3 ambiguous melanocytic proliferations which were classified as melanomas. These were 2 nevoid melanomas (patient #24 and #26) with locoregional metastasis in their further course and one nevus-associated NMM (patient #11). Of these, only the NMM showed diffuse PRAME staining (Figure 3).

### 3.2. Compound Nevi

**Figure 4:**
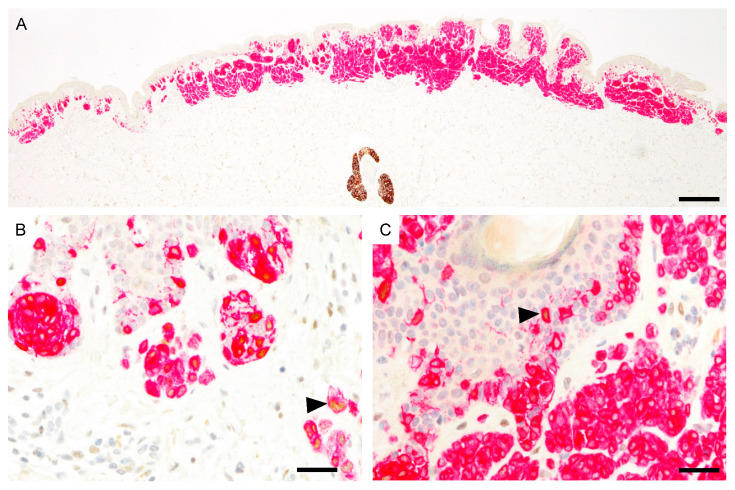
PRAME immunoreactivity in compound nevus (PRAME, brown; Melan-A, red). (**A**), Predominantly PRAME negative nevus. The sebaceous gland shows strong cytoplasmic PRAME labeling. (**B**), PRAME expression in scattered melanocytes at the lateral margin (black arrowhead). (**C**), Melanocytes in the center are predominantly PRAME negative. Single cells show PRAME immunoreactivity (black arrowhead). Abbreviation: PRAME, PReferentially expressed Antigen in MElanoma. Scale bar 500 µm (**A**), 50 µm (**B**,**C**).

The epidermal PRAME expression of unequivocally benign compound nevi ranged from 1+ to 2+ and the dermal PRAME expression from 0 to 1+. PRAME immunoreactive melanocytes were distributed very heterogeneously and were present predominantly at the lateral margins of the nevi (Figure 4).

### 3.3. Severely Dysplastic Compound Nevi (SDN)

**Figure 5:**
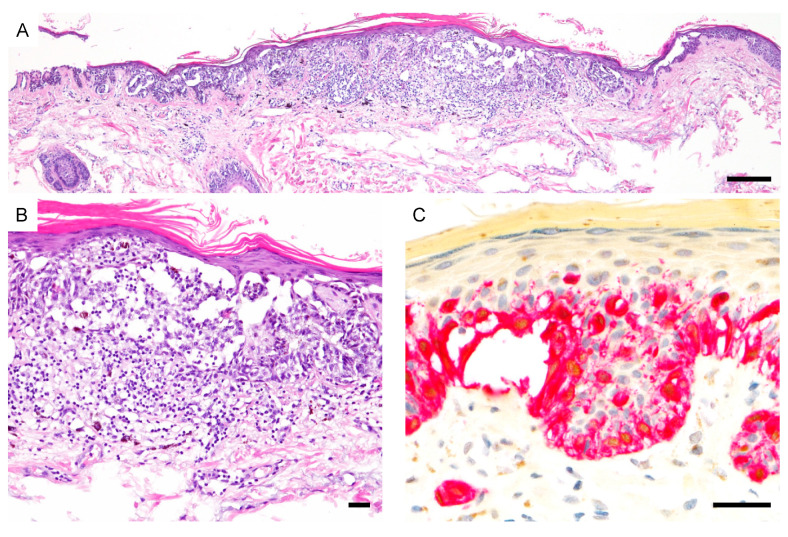
PRAME immunoreactivity in a superficial atypical melanocytic proliferation of uncertain significance (SAMPUS). (**A**,**B**), The final diagnostic interpretation favored a severely dysplastic compound nevus but an early-stage melanoma could not be excluded (H&E). (**C**), Epidermal and dermal melanocytes show diffuse PRAME expression (PRAME, brown; Melan-A, red). Abbreviation: PRAME, PReferentially expressed Antigen in MElanoma. Scale bar 250 µm (**A**), 50 µm (**B**,**C**).

**Figure 6:**
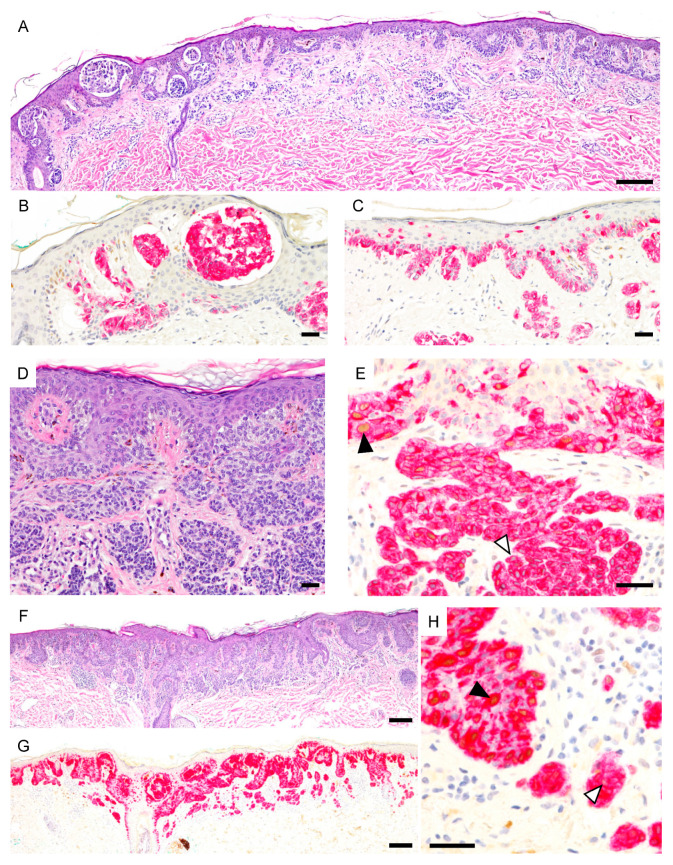
PRAME expression in severely dysplastic compound nevi. (**A**), Severely dysplastic compound nevus with irregular nests, areas of confluent growth and focal pagetoid spread (H&E). (**B**,**C**), Melanocytes are PRAME negative (PRAME, brown; Melan-A, red). (**D**). Severely dysplastic compound nevus with confluence and bridging of nests and focal pagetoid spread (H&E). (**E**), Epidermal melanocytes show focal PRAME expression (black arrowhead) but dermal melanocytes are predominantly PRAME negative (white arrowhead) (PRAME, brown; Melan-A, red). (**F**), Severely dysplastic compound nevus with confluent lentiginous pattern and focal pagetoid spread (H&E). (**G**,**H**), Epidermal melanocytes show PRAME immunoreactivity (black arrowhead) but dermal melanocytes are predominantly PRAME negative (white arrowhead) (PRAME, brown; Melan-A, red). Abbreviation: PRAME, PReferentially expressed Antigen in MElanoma.Scale bar 250 µm (**A**,**F**,**G**), 50 µm (**B**–**E**).

The mean age of the 35 patients with SDN was 45 years and ranged from 20 to 70 years. The male to female ratio was 1.3 to 1 and 13/35 patients (37.1%) had one or more invasive cutaneous melanomas. None of the patients developed metastases after a mean follow-up of 92 months (range 31–141). Table 3 summarizes the PRAME IHC results in SDN. One of 35 SDN showed diffuse PRAME staining (2.9%). This was a superficial atypical melanocytic proliferation of uncertain significance (SAMPUS), in which the final diagnostic interpretation favored a SDN, but an early-stage melanoma could not be excluded. A reexcision with 5 mm margins was recommended and follow-up of 121 months remained uneventful (Figure 5). Of the remaining 21 SDN with PRAME immunoreactivity, 81.0% showed weaker PRAME staining in the dermal than in the epidermal portion (Figure 6).

### 3.4. Summary of PRAME Immunohistochemistry

**Figure 7:**
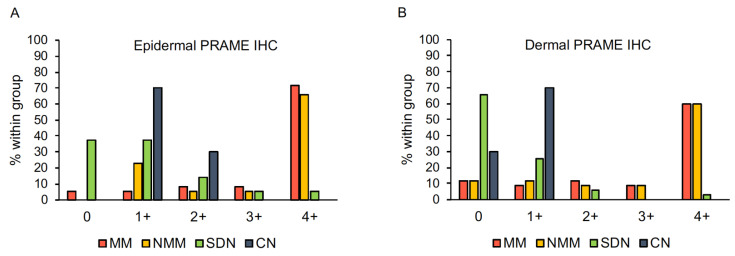
Epidermal (**A**) and dermal (**B**) PRAME expression in 115 melanocytic tumors. Abbreviations: CN, compound nevus. IHC, immunohistochemistry. MM, metastasizing melanoma. NMM, non-metastasizing melanoma. PRAME, PReferentially expressed Antigen in Melanoma. SDN, severely dysplastic compound nevus.

PRAME expression in the epidermal and dermal portions of the 70 matched melanomas and 45 nevi is summarized in Figure 7. Overall, there was a good interrater concordance regarding diffuse (PRAME score 4+) vs. non-diffuse (PRAME score from 0 to 3+) PRAME staining (κ = 0.74).

## 4. Discussion

In recent years, the CTA PRAME has received much attention in the diagnosis of melanocytic tumors following the description by Lezcano et al. that diffuse PRAME expression is highly specific and sensitive for non-spindle cell melanomas [1,5,21]. Lezcano’s work on PRAME expression in nevi and melanomas is seminal for the use of PRAME as adjunct marker in the diagnosis of melanocytic proliferations, but has some important limitations [1]:

(I) The mean Breslow thickness of the studied invasive melanomas was 3.3 mm (median 1.7 mm), which represents a clear selection towards thicker melanomas than in reality, in which the median tumor thickness ranges around 0.58 mm and melanomas ≤1.0 mm account for approximately 70% of all cases [22,23].
(II) The utility of PRAME IHC for the distinction of thin melanomas from SDN, the most common dilemma in the diagnosis of melanocytic lesions, was not studied.
(III) PRAME expression was not compared between metastasizing and non-metastasizing melanomas and the prognostic significance of PRAME in cutaneous melanomas remains to be clarified.

The aim of our study was to answer these unresolved questions by investigating PRAME expression in thin melanomas (≤1.00 mm) and SDN including ambiguous melanocytic tumors with long-term follow-up in a real-world setting.

Here, we show that diffuse PRAME expression in superficial atypical melanocytic proliferations is highly specific for melanomas. The previously reported high sensitivity of diffuse PRAME staining for thick melanomas cannot be confirmed for thin melanomas and was only 58.6% in our study [1]. The fact that the only SDN with diffuse PRAME staining was also the only case in which an early invasive melanoma could not be excluded histologically (SAMPUS) shows that diffuse PRAME expression correlates well with histological criteria for malignancy.

Non-diffuse PRAME expression by no means excludes a thin melanoma, as this staining pattern was observed in almost half of our studied melanomas. The finding that even fatal thin melanomas often demonstrate intermediate PRAME staining (scores from 1+ to 3+) and may exhibit low or even no PRAME expression in the invasive component is important, as this could lead to the misinterpretation of a nevus-associated melanoma in situ (MIS) or SDN by the unaware. Our findings recapitulate the experiences by Lohman et al., who noticed in clinical practice that “PRAME does tend to highlight MIS more diffusely than early invasive dermal components” and “more advanced melanomas tend to be equally diffusely positive as the MIS component” [5]. These shared observations suggest that there is a true difference in the staining pattern of thin and thick melanomas and that our results are not based on the use of a different PRAME clone. The awareness that PRAME IHC shows a decreasing gradient toward depth in a subset of thin melanomas is also important for tumor thickness measurement. In this regard, PRAME IHC should be used with caution and the findings correlated with those of more homogeneous immunomarkers such as Melan-A and hematoxylin and eosin staining.

Our work, together with other studies on PRAME expression in melanocytic tumors, raises the question which PRAME threshold supports a melanoma diagnosis. Lezcano et al. reported that diffuse PRAME expression in >75% of lesional melanocytes was found in around 90% of conventional melanomas (acral, superficial spreading, nodular, lentigo maligna) but only in 0.7% of nevi [1]. However, it is striking that all subsequent studies from other groups, including ours, described lower PRAME expression in cutaneous melanomas. Lohman et al. showed that PRAME staining in >76% of lesional melanocytes identified 53% of nevus-associated invasive melanomas with 100% specificity, which is comparable to our results [5]. In a study by Raghavan et al., any intensity of PRAME staining in at least 60% of lesional melanocytes discriminated best between overtly benign and malignant lesions and Gradecki et al. considered PRAME IHC positive if at least 50% of lesional melanocytes stained with PRAME [4,6].

Lowering the threshold to more than 50% PRAME positive epidermal and dermal melanocytes in our study would have resulted in a slightly better sensitivity for melanomas (67.1% vs. 58.6%) with unchanged specificity. However, a lower cut-off carries a higher risk that SDN with borderline PRAME expression will be misclassified as melanomas. This is especially true for nevi with inhomogeneous and weak PRAME expression, in which interpretation of the staining result may vary interindividually. It is also worth noting that 3+ PRAME staining was not described by Lezcano et al. in any of the investigated unequivocally benign nevi, but in diagnostically problematic benign melanocytic tumors [1,21]. These and our data suggest that a PRAME score of 4+ rather than 3+ should be preferred as a threshold for a potential melanoma diagnosis. The use of a higher cut-off is also advisable to counteract the diagnostic drift of classifying severely atypical melanocytic tumors as melanoma rather than SDN [24] and to prevent overdiagnosis of thin melanomas [18]. With the exception of spitzoid tumors, diffuse PRAME staining is virtually absent in benign melanocytic tumors [1,4,21]. Therefore, a potential nevus diagnosis should be critically questioned in atypical non-spitzoid proliferations with diffuse PRAME expression, and a complete excision of the lesion considered. However, it must be emphasized that diffuse PRAME expression does not equal malignancy and that the final diagnostic interpretation of atypical melanocytic tumors should not be based on PRAME staining alone but always on a synthesis of cytomorphological, architectural, and immunohistochemical findings. Finally, it should be noted that in our clinical practice we have rarely observed cases of non-spitzoid nevi which showed diffuse PRAME expression.

PRAME promotes the colony formation of melanoma cells in vitro [25] and tumor growth in vivo [26]. However, the prognostic role of PRAME in melanoma patients is unknown. Our study found no prognostic relevance of PRAME IHC in thin melanomas. Furthermore, the comparable proportion of melanomas with diffuse PRAME staining in the metastasizing and non-metastasizing group provided no evidence that our indolent melanomas were over-diagnosed and therefore not true melanomas.

PRAME was originally described in melanoma cell lines as a tumor antigen recognized by autologous cytotoxic T cells [27]. Its preferential expression in cancers and male germ cells in the testis but not in adult somatic tissue classifies it as a CTA [28]. The intratumoral expression of CTA, including PRAME, is highly heterogeneous and showed marked differences in different single cell clones of a melanoma cell line [29]. Here we show that this is also true to varying extents for benign and malignant melanocytic proliferations in vivo. Expression of CTA in melanomas are epigenetically regulated and suppressed by promoter hypermethylation [29]. It could be shown that hypermethylation patterns found in early disease stages gradually decrease and almost disappear in higher stages, which may explain the lower and more heterogeneous PRAME expression in thin melanomas compared to thick melanomas [30].

With regard to histological subtype, it is striking that thin melanomas with high cumulative sun damage (lentigo maligna melanoma) demonstrated more often diffuse PRAME staining than melanomas with low cumulative sun damage (superficial spreading melanoma). Lezcano et al. reported scattered PRAME positive melanocytes in non-lesional portions of lentigo maligna reexcisions and solar lentigines, indicating that in sun-damaged skin the frequency of PRAME positive melanocytes is intrinsically higher [1].

The high incidence of melanomas in our cohort with SDN (37.1%) confirms the observation that patients with dysplastic nevi are more likely to develop melanomas. Melanoma risk correlates with the severity of dysplasia and was 5.7% in patients with mild dysplastic, 8.1% with moderate, and 19.7% with severe dysplastic nevi in one study [31].

The comparability of our data with previous studies is limited by the use of a different PRAME clone (QR005). Clone QR005 yielded the best staining results in our laboratory and shows strong cytoplasmic labeling of sebaceous glands as described for clone EPR20330. Given that our staining results closely reflect the experiences and findings of other authors, however, we believe that clone QR005 has a comparable sensitivity and specificity [3,4,5,6].

## 5. Conclusions

In summary, we demonstrate that PRAME IHC is a useful adjunct in the distinction of thin melanomas from SDN. This study highlights that PRAME expression in thin melanomas is lower and more heterogeneous than has been demonstrated for in situ and thicker melanomas. The utility of diffuse PRAME staining in the group of superficial atypical melanocytic proliferations lies particularly in its high specificity for melanomas. The comparison of PRAME expression between MM and NMM showed that PRAME IHC is not a prognostic biomarker in thin melanomas.

## Figures and Tables

**Table 1:** Comparison of PRAME immunohistochemistry in matched metastasizing and non-metastasizing thin melanomas ≤1.0 mm.

		Metastasizing Melanomas						Non-Metastasizing Melanomas				
Patient No./Sex	Site	Age (y)	Depth (mm)	PRAME IHC		Stage	FU (mo) and Outcome	Age (y)	Depth (mm)	PRAME IHC		FU (mo)
				Epidermis	Dermis					Epidermis	Dermis	
1/F	T	52	0.8	1+	0	III	93/A	48	0.8	4+	4+	88
2/F	T	64	0.7	4+	4+	III	68/A	69	0.6	1+	1+	93
3/M	T	59	0.4	1+	0	IV	9/D	54	0.4	1+	0	133
4/M	T	49	0.5	2+	1+	IV	91/D	49	0.4	4+	4+	153
5/M	H&N	54	0.5	4+	4+	IV	139/A	61	0.5	4+	3+	123
6/F	LE	67	0.6	4+	4+	IV	86/D	70	0.6	4+	4+	117
7/M	LE	60	0.6	4+	4+	IV	103/A	65	0.7	4+	2+	146
8/M	H&N	80	0.4	4+	3+	IV	70/D	80	0.4	4+	4+	84
9/M	LE	61	0.4	4+	4+	IV	80/A	65	0.5	1+	0	123
10/M	LE	35	0.7	4+	4+	IV	30/D	45	0.6	1+	1+	121
11/M	T	31	0.3	4+	4+	IV	5/D	36	0.4	4+	4+	110
12/M	H&N	32	0.6	4+	4+	IV	121/A	30	0.5	2+	2+	51
13/M	T	70	0.6	3+	2+	IV	26/A	76	0.6	1+	0	134
14/F	H&N	54	0.6	3+	2+	IV	84/D	55	0.6	4+	4+	74
15/M	T	79	0.7	0	0	IV	73/D	72	0.7	4+	4+	116
16M	H&N	91	0.8	4+	4+	IV	14/A	81	0.7	4+	4+	53
17/F	T	61	0.8	4+	3+	IV	6/D	56	0.9	1+	1+	118
18/M	H&N	87	0.5	4+	4+	IV	17/A	78	0.5	4+	3+	142
19/F	H&N	77	0.8	4+	4+	III	43/A	68	0.7	4+	4+	91
20/M	H&N	90	0.8	4+	4+	IV	75/D	86	0.8	4+	4+	31
21/F	LE	41	0.8	2+	1+	IV	76/D	35	0.9	4+	4+	63
22/M	UE	53	0.8	4+	4+	III	42/A	61	0.9	4+	4+	50
23/F	LE	66	0.8	4+	4+	IV	129/A	60	0.9	4+	4+	104
24/F	UE	36	0.9	4+	2+	III	38/A	42	0.8	3+	4+	113
25/M	H&N	72	0.9	4+	4+	IV	86/A	69	0.8	4+	4+	129
26/F	UE	62	0.9	2+	1+	III	75/A	67	0.8	4+	4+	96
27/F	T	54	0.9	4+	4+	III	109/A	56	0.9	1+	0	118
28/F	T	31	0.9	0	0	III	17/A	37	0.9	4+	4+	64
29/M	T	66	0.9	3+	2+	III	63/A	70	0.9	1+	1+	85
30/M	H&N	76	0.9	4+	4+	III	9/A	75	1.0	4+	4+	67
31/M	T	64	0.9	4+	3+	III	114/D	61	0.9	2+	3+	123
32/M	T	59	1.0	4+	4+	III	71/A	60	1.0	4+	4+	124
33/M	UE	72	1.0	4+	4+	IV	88/A	71	1.0	4+	4+	57
34/F	LE	66	1.0	4+	4+	IV	87/D	60	1.0	3+	2+	110
35/F	T	87	0.9	4+	4+	III	31/A	83	1.0	4+	4+	54

Abbreviations: A, alive. D, died of melanoma. F, female. FU, follow-up. H&N, head and neck. IHC, immunohistochemistry. LE, lower extremity. M, male. mo, months. PRAME, PReferentially expressed Antigen in MElanoma. T, trunk. UE, upper extremity. y, years.

**Table 2:** Summary of PRAME expression in 115 melanocytic tumors.

Histological Diagnosis and Characteristics	Cases with Diffuse PRAME Expression ^†^	*p*-Value
Nevus		
Compound nevus	0/10	
Severely dysplastic compound nevus	1/35 (2.9%) ^‡^	<0.001
Melanoma	41/70 (58.6%)	
Metastasizing	21/35 (60.0%)	0.81
Non-metastasizing	20/35 (57.1%)	
Subtype		
Superficial spreading	26/48 (54.2%)	0.074
Lentigo maligna	12/16 (75.0%)	
Acral lentiginous	3/3 (100%)	
Nevoid	0/2 (0%)	
Spitzoid	0/1 (0)	
Regression		
Present	17/33 (51.5%)	0.26
Absent	24/37 (64.9%)	
TNM		
pT1a	17/31 (54.8%)	0.57
pT1b	24/39 (61.5%)	

^†^ PRAME immunoreactivity in >75% of epidermal and dermal lesional melanocytes was regarded diffuse. ^‡^ In the case with diffuse PRAME staining, the final diagnostic interpretation favored a severely dysplastic nevus, but an early-stage melanoma could not be excluded, corresponding to a superficial atypical melanocytic proliferation of uncertain significance (SAMPUS). Abbreviation: PRAME, PReferentially expressed Antigen in MElanoma.

**Table 3:** Characteristics and PRAME immunohistochemistry of 35 severely dysplastic compound nevi.

Patient No./Sex.	Site	Age (y)	PRAME IHC		Known Invasive Melanoma	FU (mo)
			Epidermis	Dermis		
1/M	T	38	0	0	−	113
2/M	T	56	0	0	+	76
3/F	LE	48	1+	0	−	54
4/M	T	42	0	0	−	126
5/F	T	46	0	0	−	45
6/F	T	50	0	0	−	71
7/F	T	20	0	0	+	141
8/M	LE	33	2+	1+	−	70
9/M	T	66	2+	2+	+	118
10/F	H&N	41	1+	0	−	48
11/F	T	45	0	0	−	105
12/F	T	42	1+	1+	−	138
13/M	T	49	1+	0	−	88
14/F	T	34	0	0	+	31
15/M	T	47	1+	0	+	112
16M	T	56	4+	1+	−	125
17/M	LE	44	3+	1+	+	93
18/M	T	62	3+	2+	−	126
19/M	T	32	0	0	−	47
20/M	T	65	1+	1+	−	78
21/M	T	51	1+	0	+	98
22/F	LE	63	1+	1+	+	132
23/F	T	50	1+	0	−	72
24/M	T	38	0	0	−	74
25/M	T	34	0	0	−	96
26/M	LE	26	1+	0	−	64
27/F	T	42	2+	1+	−	114
28/F	UE	32	2+	1+	−	81
29/M	T	61	1+	0	+	45
30/M	LE	28	0	0	+	80
31/F	T	28	1+	0	−	95
32/M	T	40	1+	0	+	116
33/F	LE	62	2+	1+	+	129
34/M ^†^	H&N	70	4+	4+	+	121
35/F	T	25	0	0	−	99

^†^ In this case, the final diagnostic interpretation favored a severely dysplastic nevus but an early-stage melanoma could not be excluded, corresponding to a superficial atypical melanocytic proliferation of uncertain significance (SAMPUS). Abbreviations: F, female. FU, follow-up. H&N, head and neck. IHC, immunohistochemistry. LE, lower extremity. M, male. mo, months. PRAME, PReferentially expressed Antigen in MElanoma. T, trunk. UE, upper extremity. y, years.

## Data Availability

The data presented in this study are available in the article and supplementary material.

## Supplementary Materials

The following are available online at https://www.mdpi.com/article/10.3390/cancers13153864/s1, Table S1: Clinicopathologic characteristics of the cases.
