# Supplementary material for: Diffuse PRAME Expression Is Highly Specific for Thin Melanomas in the Distinction from Severely Dysplastic Nevi but Does Not Distinguish Metastasizing from Non-Metastasizing Thin Melanomas
Source: Cancers (Basel). 2021 Jul 31;13(15):3864. doi: 10.3390/cancers13153864 (PMC8345662; doi:10.3390/cancers13153864)
Supplement: Supplementary file 1 [file cancers-13-03864-s001.zip › cancers-1260056-supplementary.pdf]

cancers

MDPI

Supplementary Material: Diffuse PRAME expression is highly specific for thin melanomas in the distinction from severely dysplastic nevi but does not distinguish metastasizing from non-metastasizing thin melanomas.

Maximilian Gassenmaier, Matthias Hahn, Gisela Metzler, Jürgen Bauer, Amir Sadegh Yazdi, Ulrike Keim, Claus Garbe, Nikolaus Benjamin Wagner and Stephan Forchhammer

Table S1. Clinicopathologic characteristics of the cases.

Characteristics	Metastasizing Melanoma (*n* = 35) n (%)	Non-Metastasizing Melanoma (*n* = 35) n (%)	Severely Dysplastic Compound Nevus (*n* = 35) n (%)	*p*-Value MM vs. NMM	*p*-Value SDN vs. Melanoma
Breslow thickness (mean, range)	0.7 mm (0.3–1.0)	0.7 mm (0.4–1.0)	n.a.	0.91	n.a.
Age (mean, range)	62 years (31–91)	61 years (30–86)	45 years (20–70)	0.95	<0.001
Gender					
Female	14 (40.0%)	14 (40.0%)	15 (42.9%)	1.0	0.84
Male	21 (60.0%)	21 (60.0%)	20 (57.1%)		
Site					
Head and neck	10 (28.6%)	10 (28.6%)	2 (5.7%)	1.0	0.005
Trunk	14 (11.4%)	14 (11.4%)	25 (71.4%)		
Upper extremities	4 (11.4%)	4 (11.4%)	1 (2.9%)		
Lower extremities	7 (20%)	7 (20%)	7 (20%)		
Subtype					
Superficial spreading	25 (71.4%)	23 (65.7%)	n.a.	0.42	n.a.
Lentigo maligna	6 (17.1%)	10 (28.6%)	n.a.		
Acral lentiginous	2 (5.7%)	1 (2.9%)	n.a.		
Nevoid	2 (5.7%)	0 (0%)	n.a.		
Spitzoid	0 (0%)	1 (2.9%)	n.a.		
Regression					
Present	18 (51.4%)	15 (42.9%)	0 (0%)	0.63	<0.001
Absent	17 (48.6%)	20 (57.1%)	35 (100%)		
Ulceration					
Present	4 (11.4%)	2 (5.7%)	0 (0%)	0.67	0.18
Absent	31 (88.6%)	33 (94.3%)	35 (100%)		
Stage					
III	14 (40.0%)	0 (0%)	n.a.	<0.001	n.a.
IV	21 (60.0%)	0 (0%)	n.a.		
Follow-up (mean, range)	65 months (5–139)	99 months (31–153)	92 months (31–141)	<0.001	0.23

Abbreviations: MM, metastasizing melanoma. n.a., not applicable. NMM, non-metastasizing melanoma. SDN, severely dysplastic compound nevus.
